# COVID-19 mortality rate in children is U-shaped

**DOI:** 10.18632/aging.203442

**Published:** 2021-08-18

**Authors:** Nina Khera, Didac Santesmasses, Csaba Kerepesi, Vadim N. Gladyshev

**Affiliations:** 1 Buckingham Browne and Nichols School, Cambridge, MA 02138, USA; 2 Division of Genetics, Department of Medicine, Brigham and Women’s Hospital and Harvard Medical School, Boston, MA 02115, USA; 3 Biotein, Wellesley, MA 02482, USA

**Keywords:** mortality, COVID-19, SARS-CoV-2, pediatrics, aging

## Abstract

Children are known to be better protected from COVID-19 than adults, but their susceptibility patterns and the risk relative to other diseases are insufficiently defined. Here, we found that the COVID-19 mortality rate is U-shaped in childhood: it initially decreases, reaching the minimum at the ages 3-10 years, and then increases throughout life. All-cause mortality and mortality from other diseases, such as pneumonia and influenza, show a similar pattern; however, childhood mortality rates from COVID-19 are considerably lower than from other diseases, with the best relative protection achieved at the youngest ages. Consistent with this, the fraction of COVID-19 deaths among all deaths increases as a function of age throughout childhood and the entire life. We discuss implications of the elevated postnatal COVID-19 risk and lower childhood COVID-19 mortality compared to other diseases.

## INTRODUCTION

Severe acute respiratory syndrome coronavirus 2 (SARS-CoV-2) is a recently emerged coronavirus that causes the ongoing COVID-19 pandemic, originating from Wuhan, China, in December 2019 [1]. COVID-19 symptoms include coughing, shortness of breath, fever, fatigue, muscle aches, and more [2], but it targets multiple organs and is aggravated by diverse comorbidities. With nearly 200 million cases worldwide and more than four million deaths, COVID-19 remains a major public health threat.

COVID-19 primarily targets older individuals and those with comorbidities, whereas younger individuals are often spared [3]. Fewer children than adults develop severe pneumonia, and exhibit inflammatory markers, and many children infected by SARS-CoV-2 show no symptoms at all [3]. It was suggested that lower burden of COVID-19 in children [4] is due to decreased severity of infection, as is the case of several other infectious diseases, such as paralytic polio, rubella and severe respiratory distress syndrome (SARS) [5]. Interestingly, in newborns, COVID-19 may develop symptomatically, but vertical intrauterine transmission is rare [3].

Human all-cause mortality rate increases exponentially, with a doubling time of around 8 years [6]. However, the trend is opposite in early life where the mortality rate decreases from birth until the age of 9, where it reaches minimum, and then increases exponentially [7]. It is unclear how COVID-19 mortality changes in early life, whether it has a minimum, and whether children are better or worse protected from COVID-19 relative to other diseases. To fill this gap, we examined COVID-19 mortality data, revealing a U-shaped pattern of COVID-19 mortality and a considerably lower severity of this disease compared to other diseases in children.

## RESULTS

### U-shaped pattern of COVID-19 mortality rate

**Figure 1:**
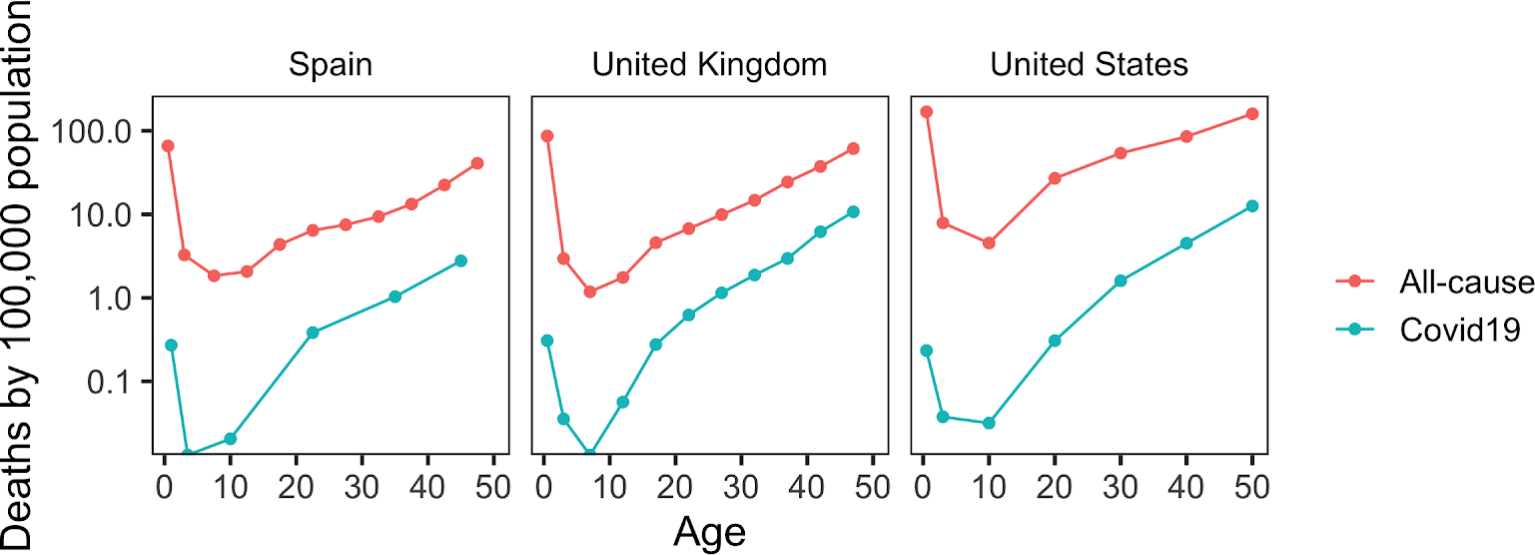
**Age-related patterns of COVID-19 mortality.** Data are shown for the indicated countries. Age-related change in all-cause mortality are also shown for these countries.

**Figure 2:**
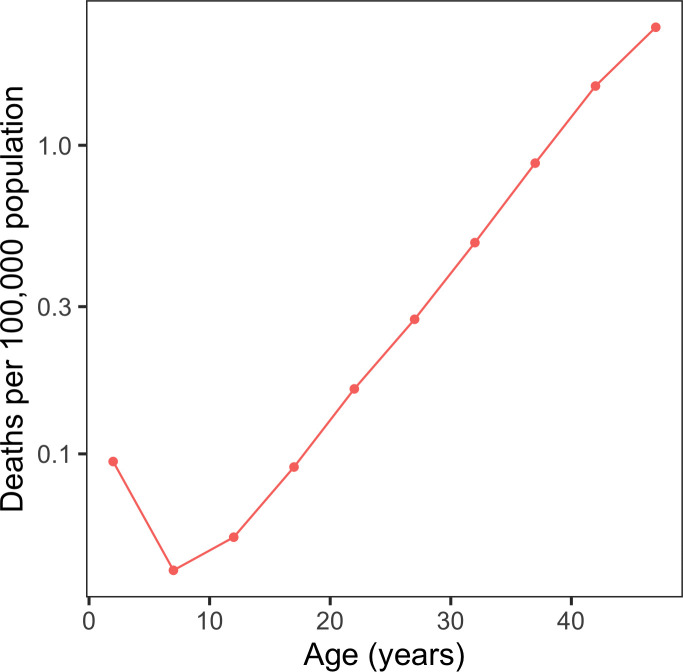
**COVID-19 deaths per 100,000 population in the world.** Shown is the world’s COVID-19 mortality rate per 100,000 people per age interval.

To understand how COVID-19 mortality changes over the entire lifespan, we analyzed the age-associated mortality rate from COVID-19 in several countries (United States, United Kingdom and Spain), and compared it to the corresponding all-cause mortality. Age-associated COVID-19 mortality rate exhibited a U-shaped pattern, with the lowest rate observed at 3-10 years of age (Figure 1). A similar pattern was observed when the analysis was done for the entire world (Figure 2). As the absolute number of deceased subjects during early life is low and reporting patterns differed across countries, it was not possible to define the exact age at the minimum. However, all examined countries showed the U-shaped pattern, with the COVID-19 mortality rate in newborns and infants typically being as high as in 20-year-old subjects. All-cause mortality showed a similar U-shaped curve, with the minimum at the age of 9; however, the difference between COVID-19 mortality and all-cause mortality was considerably higher in young ages.

### COVID-19 mortality shows the lowest rate in childhood compared to other related diseases

**Figure 3:**
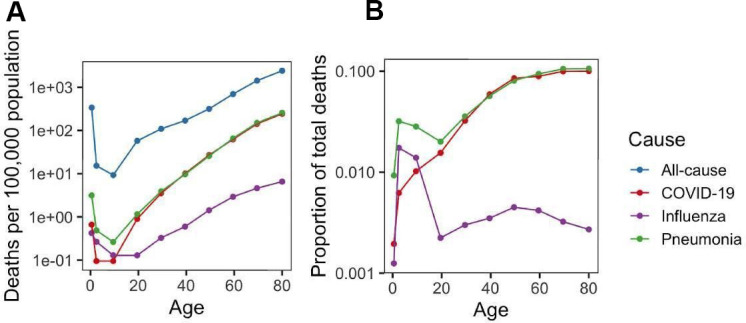
**Burden of COVID-19, pneumonia and influenza.** (**A**) Mortality rate (per 100,000 population) for COVID-19, pneumonia and influenza as well as all-cause mortality rate. (**B**) Ratio of COVID-19, pneumonia and influenza mortality rate to all-cause mortality rate.

We analyzed childhood mortality rates for other diseases in the US and compared them to that of COVID-19 (Figure 3). Like COVID-19, influenza and pneumonia showed a U-shaped pattern that paralleled all-cause mortality. However, the COVID-19 mortality rate was disproportionally low in children under 12 (Figure 3A), suggesting a better protection against severe COVID-19 at the young ages compared to the two other infectious diseases. This could also be seen by the proportion of total deaths, which spiked at very early ages for pneumonia and influenza, but not for COVID-19 (Figure 3B). In adults over 20 years, the proportion of total deaths increased steadily with age for COVID-19 and pneumonia and then reached a plateau around 10% of all deaths. Interestingly, influenza showed a very different pattern, with a stable proportion well below 1% that did not increase with age.

**Figure 4:**
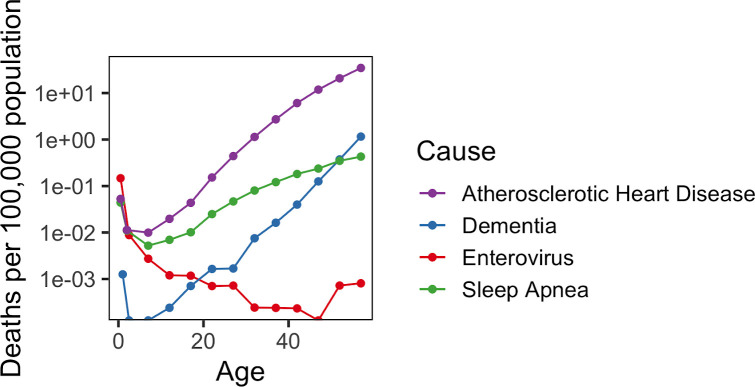
**Mortality rate from other diseases.** Mortality rate (per 100,000 population) across lifespan is shown for atherosclerotic heart disease, dementia, sleep apnea, and enteroviral infection.

Considering the common U-shaped mortality curves in early life for all analyzed diseases as well as for all-cause mortality, age emerged as an important risk factor for COVID-19 mortality from birth until the end of life. Additionally, we examined mortality rate from unspecified dementias, enteroviral infection, atherosclerotic heart disease, and sleep apnea (Figure 4). Their mortality patterns were also U-shaped, except for enteroviral infection, which had the highest mortality rate after birth and then decreased with age. These diseases were chosen to represent broader categories: enterovirus to cover the immune system and response [8], dementia to cover the nervous system, atherosclerosis and COVID-19 to cover the vascular system [9], and sleep apnea to cover the respiratory system. Even though enterovirus and the novel coronavirus represent infectious diseases, they exhibited remarkably different patterns, with enterovirus having the mortality rate minimum at the age of 40.

### Children exhibit lower rates of severe symptoms than adults

**Figure 5:**
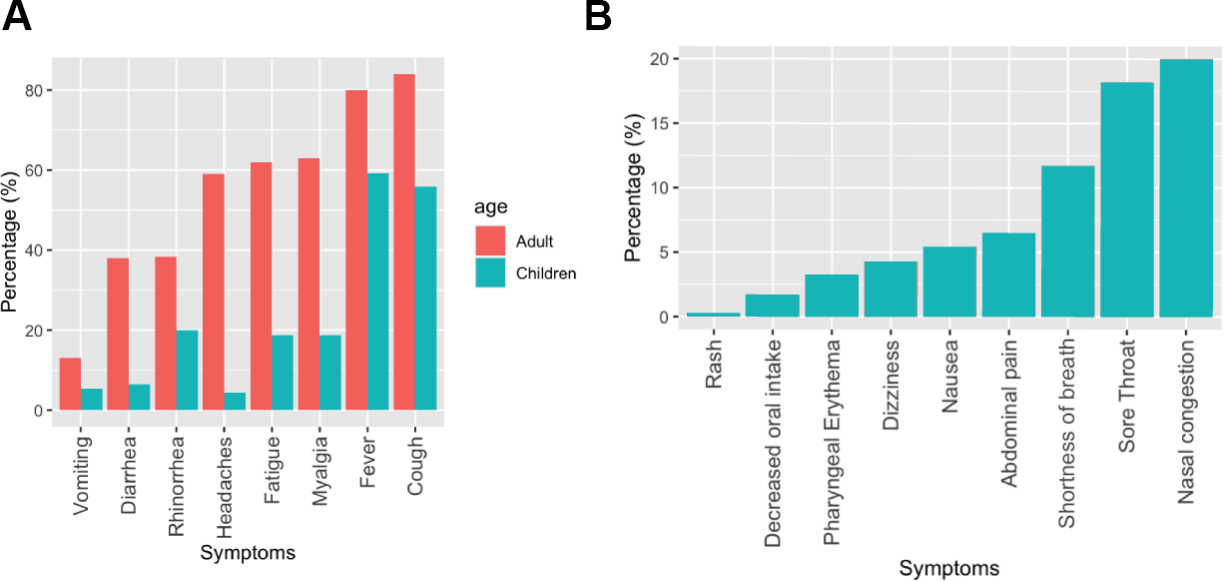
**Frequency of COVID-19 symptoms in children compared to adults.** (**A**) Frequency of common COVID-19 symptoms in children and adults. (**B**) Rank order of COVID-19 symptoms specific to children.

We examined COVID-19 symptoms in children compared to adults (Table 1 and Figure 5), taking advantage of previously collected datasets [10, 11]. Adults showed higher rates of common symptoms than children, further supporting the protection that young ages offer against this disease. For example, 80% of adults had fever, compared to 59.9% of children, 84% of adults had cough versus 55.9% of children, and 38.4% of adults had rhinorrhea compared to 20% of children. Children also had unique symptoms in these studies, with nasal congestion being most frequent (20%), followed by sore throat (18.2%), and shortness of breath (11.7%) (Figure 5B). Some of these symptoms could be seen in adults in other studies, in particular, adults may experience shortness of breath during COVID-19 [12]. Additionally, there have been reports of adults having sore throat [13].

**Table 1:** Frequency of COVID-19 symptoms in US children and adults.

**Symptoms**	**Children in the US (%)**	**Adults in the US (%)**
Fever of at least 37.5 degrees	59.1	80
Cough	55.9	84
Rhinorrhea	20	38.4
Nasal congestion	20	N/A
Fatigue	18.7	62
Myalgia	18.7	63
Sore Throat	18.2	N/A
		
Shortness of breath	11.7	N/A
Diarrhea	6.5	38
Abdominal pain	6.5	N/A
Nausea	5.4	N/A
Vomiting	5.4	13
Headaches	4.3	59
Dizziness	4.3	N/A
Pharyngeal Erythema	3.3	N/A
Decreased oral intake	1.7	N/A
Rash	0.25	N/A
Chills	N/A	63
Tachypnoea	N/A	57
Changes in smell or taste	N/A	13.9

## DISCUSSION

We found that COVID-19 shows a U-shaped pattern of age-associated mortality in several analyzed countries. The risk of dying from COVID-19 decreases during early life up to the age of 3-10 years, where it reaches its minimum, and then increases exponentially throughout life. The lowest rates were observed in 7-9 years old children in the US and UK, and 3-4 years old in Spain, but the exact minimum was difficult to pinpoint due to few cases of mortality at these ages. A similar pattern was found when we analyzed COVID-19 deaths across 37 countries based on COVerAGE-DB [14] (Figure 2). Although it is commonly believed that children are completely spared of COVID-19, our findings suggest that newborns and children during their first year of life exhibit a slightly elevated COVID-19 risk. Moreover, although it was not analyzed in this study, fetal mortality rate may be expected to be even higher. This is because COVID-19 follows a characteristic U-shaped mortality curve, also observed in the case of all-cause mortality and many diseases.

Our second important finding is that children are disproportionately better protected from COVID-19 than from other common diseases analyzed in the study. While pneumonia and influenza also exhibited a U-shaped pattern, COVID-19 showed a lower mortality rate in children below the age of 12. COVID-19 mortality rate has previously been associated with aging [15], and analyses of infection rates [5] revealed a lower susceptibility in children. However, our study shows that COVID-19 susceptibility is not just lower in children than adults, but lower relative to many other diseases.

Some studies, such as a multinational cohort study in Europe [16], analyzed children with COVID-19 admitted to hospitals, and their observed patterns showed parallels with our results. The highest rate of ICU admission corresponded to the first month after birth, and the case-fatality rate for children was on average 0.69%. The most common source of infection was a parent or sibling.

Models that may help explain our findings are related to differences in the expression of ACE2, the SARS-CoV-2 receptor [17], and the lower burden of COVID-19 symptoms in children [3]. Children were found to express lower levels of ACE2 [17], while having a robust innate immune system to better deal with the virus during the entry point to the organism. Also, children may have had fewer opportunities to be infected due to the public health measures during the pandemic, such as school closures. It was also theorized that the impaired immune response could lead to higher COVID-19 vulnerability in adults [18], with older adults having a greater risk of mortality from infectious diseases that are targeted by common vaccines [19]. All these factors may lead to a much lower severity of COVID-19 in children except for newborns [10, 11]. Differences in COVID-19 symptoms may also related to gene expression patterns. The expression of genes IFNAR2 and TYK2 was found to be related to critical illness and severity of COVID-19 symptoms [20]. IFNAR2 is involved in innate antiviral defense, which is known to be important early in COVID-19. TYK2 is involved in host-driven inflammatory lung injury, which may lead to more severe symptoms [20, 21].

Another study analyzed disease severity and symptoms amongst children versus adults in China [21] and found that the majority (66.7%) of children in Jinan Infectious Diseases Hospital exhibited no symptoms. None of the children in the study needed a ventilator or additional accommodations. However, it reported a high level of creatinine kinase-MB (CK-MB; an indicator of myocardial injury) in most children versus few adults, suggesting that children may exhibit higher rates of myocardial injury caused by the virus. Our analyses also revealed symptoms that were more common in children. The symptoms common to both children and adults were generally less severe in children. An additional study that examined symptoms in children had similar findings [22], e.g. it was found that respiratory symptoms were generally mild, with fever being the most common symptom amongst children.

**Figure 6:**
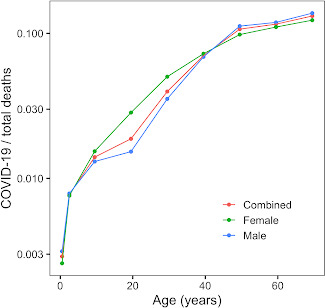
**Fraction of total deaths in the United States attributable to COVID-19.** Fraction of total deaths, sorted by age and color-coded by sex, of total recorded deaths in the United States that had their cause pinpointed as COVID-19.

Limitations of our study include the fact that it was restricted to a relatively small number of COVID-19 cases in children due to the novelty of the disease. As we did not consider ethnicity or gender, additional studies are needed to address their relevance [23]. Along these lines, we examined the fraction of total deaths related to COVID-19 in the US separately for the two sexes (Figure 6). The most prominent difference was around the age 20, which is consistent with riskier behavior by men at these ages. An interesting area to explore is the impact of a previous COVID-19 infection on children’s overall health and long-term wellness. A case report of 5 COVID-19 cases in children in Sweden showed that 6-8 months after infection, they stopped experiencing most of the symptoms, but their fatigue persisted [24].

Overall, our study establishes that the COVID-19 mortality rate is U-shaped, like that of pneumonia, influenza and many other diseases. Thus, newborns have a somewhat increased risk of severe COVID-19, and children below the ages 1-3 years exhibit elevated COVID-19 burden compared to older children. On the other hand, children in general are greatly spared from COVID-19 compared to other common diseases. These pronounced age-related patterns suggest that the factors that affect biological age may influence COVID-19 infection and severity. Thus, while COVID-19 is known to be most dangerous for the elderly and those with chronic diseases, children are also affected, with the perinatal period being more dangerous that later childhood. Finally, our study suggests the observed age-related patterns of COVID-19 susceptibility in children should be considered when developing COVID-19 regulations.

## MATERIALS AND METHODS

### Data sources

United States data on confirmed COVID-19 cases and hospitalizations categorized by age were from the Center for Disease Control [25]. We used the Office for National Statistics’ data on all-cause mortality, reported weekly [26], as well as their data on the population in different regions of the UK by age [27]. These were used for the analysis of fatality rates in the US and the UK. As a reference, we used statistics of ‘Our world in data’ on all-cause infant and child mortality [28]. We computed deaths per 100,000 population. For the same analysis with Spain, we used Insitituto de Salud Carlos III’s data on COVID-19 cases and fatalities [29]. Data from the United Nations on population was used to inform many of the population-related calculations [30]. Additionally, data from hospitals was used to comparatively analyze symptomal frequencies [10, 11]. The data was last searched December of 2020. There is no review protocol.

### Calculation of COVID-19 mortality rates

Total COVID-19 deaths by 100,000 population were calculated for Spain, the UK, and USA using data on COVID-19 deaths overall and population from various governmental centers and hospitals [25–27, 29], and plotted using ggplot in R. COVID-19 case-fatality ratios per 100,000 people were calculated based on COVerAGE [14] and plotted using ggplot in R.

### Comparison of mortality from COVID-19, pneumonia and influenza

Mortality rate was calculated for different diseases, as well as all-cause mortality rate, and the ratios of the mortality rates from the aforementioned diseases to all-cause mortality rate were computed. This was done using data on deaths from these different diseases, all-cause deaths, and population within the US from the CDC and the government overall [25, 30].

### Mortality from other diseases

Mortality rate was calculated for various diseases, as well as all-cause mortality rate, and the ratios of the mortality rates from the aforementioned diseases to all-cause mortality rate were computed. This was done using data on deaths from these diseases, all-cause deaths, and population within the US from the CDC Wonder Database [31].

### Analysis of COVID-19 symptoms in children compared to adults

Multiple studies of percentages of symptoms in children and adults were examined, and statistics that were found per symptom were plotted, comparing both children and adults, and children overall. The data was retrieved from official hospital studies [10, 11]. Everything was plotted using ggplot in R. For Table 1, the aforementioned symptomal statistics were added. In some datasets, data was presented as part of different weeks. When this was the case, all relevant data was added and compiled.
